# Hypophosphatasia and Type 1 Diabetes: A Pilot Study and Review of Literature

**DOI:** 10.1007/s11914-025-00938-x

**Published:** 2025-10-09

**Authors:** Zunera Tariq, Jake P. Tinsley, Dana Carpenter, Janet K. Snell-Bergeon, Viral N. Shah

**Affiliations:** 1 Division of Endocrinology & Metabolism, Indiana University School of Medicine, Indianapolis, IN USA; 2 Department of Mechanical Engineering, University of Colorado Denver, Denver, CO USA; 3 Barbara Davis Center for Diabetes, University of Colorado Anschutz Medical Campus, Aurora, CO USA

**Keywords:** Hypophosphatasia (HPP), Fracture risk, Type 1 diabetes (T1D), Finite element (FE) analysis, Bone mineral density (BMD), Quantitative CT (QCT)

## Abstract

**Purpose of Review:**

Hypophosphatasia (HPP) and type 1 diabetes (T1D) are both associated with low bone turnover and increased fracture risk. In this review, our objectives were to (a) discuss results of our pilot study aimed to evaluate prevalence and clinical and radiological characteristics of HPP in adults with T1D and (b) to review literature on the use of electronic medical records (EMR) for HPP case findings.

**Recent Findings:**

In our pilot study, 18 individuals had persistent low alkaline phosphatase levels (ALP) [18/1723, 1.05%]. Among 10 participants who completed the study with potential HPP and T1D, three had a pathogenic ALPL mutation (0.16% prevalence), and six had elevated serum vitamin B6. No significant differences were found in DXA-based bone density, QCT-based bone density, or Finite element-estimated bone strength between the potential HPP group, T1D adults, and controls. We did not find any study that evaluated persistent low ALP levels or potential HPP in patients with diabetes (either type 1 or type 2 diabetes). The literature reported higher prevalence of low ALP levels when electronic medical records were searched (~ 1–3%). However, prevalence of suspected HPP based on persistent low ALP levels and/or clinical signs and symptoms was around 0.5% or lower depending on sample size, methods and geographical locations.

**Summary:**

Our study suggests that EMR-based screening for HPP is feasible and may identify previously undiagnosed cases of HPP. Prevalence of potential HPP in T1D is around 1% and genetically confirmed HPP is 0.16% which is similar to reported prevalence of HPP in selected population without diabetes. Skeletal imaging and clinical presentations are not sufficient for identifying potential HPP in individuals with T1D. Given the increased fracture risk and low bone turnover typically seen in T1D, we propose that an EMR-based screening strategy could be a more effective approach for diagnosing HPP in T1D population.

**Supplementary Information:**

The online version contains supplementary material available at 10.1007/s11914-025-00938-x.

## Introduction

Hypophosphatasia (HPP) is a metabolic disorder caused by a loss of function mutation in the ALPL gene, leading to reduced tissue activity of alkaline phosphatase (ALP) [1]. The prevalence of HPP is estimated to be around 0.01%−0.06% in the European population [2, 3]. Since most cases of adult onset HPP occur with minimal symptoms, and remain undiagnosed, systematic electronic medical record (EMR) search has been reported to yield a higher prevalence of HPP, around 0.2% among adults presenting with metatarsal stress fracture [4].

Low serum ALP levels (in the absence of other causes) are generally considered a hallmark of HPP and associated with increased fracture burden^1^. It is important to remember that low ALP levels are commonly encountered in many conditions such as nutritional deficiencies (zinc, magnesium or B6 or B12 deficiency), use of anti-resorptive medications, endocrine conditions and preanalytical laboratory errors (these factors are detailed in reference 41). Hence, it is important to exclude all potential secondary causes of low ALP levels before suspecting HPP. Studies have also reported low bone formation markers in patients with HPP [5]. Type 1 diabetes (T1D) is also a disease of low bone turnover, which has been implicated in increased fracture risk observed in this population [6–8]. Most but not all, studies have reported lower bone formation including lower serum ALP (most reporting low bone specific ALP) in people with T1D [9, 10]. Hence, a low ALP level in individuals with T1D, even in the presence of a fracture, may not alert clinicians to the possibility of HPP, making its diagnosis more challenging.

To our best knowledge, prevalence of HPP comorbidity in adults with T1D and its potentially cumulative effects on skeletal health has not been studied. We undertook a pilot study with two main objectives: (a) to estimate the prevalence of potential adult-onset HPP in individuals with T1D, and (b) to compare clinical and radiological features among individuals with T1D and potential HPP, individuals with T1D without HPP, and individuals without diabetes. We also reviewed literature to evaluate prevalence of low ALP levels and potential HPP diagnosis when EMR is being employed as a potential case finding strategy.

## Methods

### Methods for a Pilot Study

#### Study Design

This was a single center, cross-sectional study approved by the Colorado Multiple Institutional Reviewer Board. First, we searched the electronic medical records (EMR) of the Barbara Davis Center for Diabetes Adult Clinic (EPIC) to estimate the number of adults with T1D who had at least two serum ALP levels below 35 U/L. This threshold was chosen to enhance the specificity of identifying HPP cases. The selection of adults with T1D and EMR search methods has been described in detail previously [11]. We excluded patients who were pregnant or had low ALP levels measured during major illness, surgery, malignancy, chemotherapy, bisphosphonate use, or vitamin D excess.

#### Study Population

The study population consisted of three groups: Group 1, adults with T1D and potential HPP (defined by at least two serum ALP levels < 35 U/L from EMR records and exclusion of secondary causes of low ALP levels); Group 2, adults with T1D and normal ALP levels; and Group 3, adults without diabetes. Participants in Group 2 and 3 were recruited from an ongoing NIH funded study (R01DK122554) aimed at evaluating bone health in adults with T1D. We recruited participants in Group 1 first and then a similar number of participants were recruited for Group 2 and Group 3 to match sample size between the three groups. T1D was defined by meeting at least one of the following criteria: (a) diagnosis between 6 months and 10 years of age, (b) positive pancreatic autoantibodies at any time (GAD-65, IA-2, ICA, or ZnT8), or clinical indicators suggestive of T1D, such as: (c) diagnosis under 30 years of age, (d) initiation of insulin therapy at diagnosis, or (e) a clinical course consistent with T1D [12]. Healthy participants without diabetes were defined as those with an HbA1c < 5.7% and no history of diabetes or use of diabetes medications [13]. Since participants in Group 2 and 3 were recruited from an ongoing R01 (R01DK122554), the additional exclusion criteria in ongoing R01 were: history of malabsorption syndrome, rheumatologic disease, parathyroid disease, cancer (other than skin cancer), long-term steroid use (oral or injectable for more than three months), immunosuppressant use in the past two years, an estimated glomerular filtration rate (eGFR) < 30 mL/min/1.73 m^2^, required dialysis, or a history of renal transplant, use of osteoanabolic or antiresorptive medications for osteoporosis treatment, or who had a history of untreated celiac disease (indicated by elevated IgA tissue transglutaminase) or uncontrolled thyroid disease (defined by thyroid-stimulating hormone levels < 0.4 or > 4.4 mIU/mL).

#### Study Procedures

After written informed consent, participants in Group 1 underwent a fasting blood test for total ALP, B6 level (after stopping their vitamin supplement(s) for at least 1 week prior to screening visit) and genetic testing for HPP. Total ALP (normal reference range for both sexes over 18 years of age; 39–117 U/L) was measured at UC Health Laboratory, Aurora, Colorado, and vitamin B6 (normal reference range 20–125 nmol/L) was estimated at ARUP laboratory, Salt Lake City, Utah. ALPL genetic sequencing was performed at Prevention Genetics (https://www.preventiongenetics.com/testInfo?val=Hypophosphatasia-via-the-ALPL-Gene). Participants in Group 1 also completed HPP disease onset, sign and symptoms (as used in HPP global registry) as described previously [14, 15]. Participants in all groups completed standardized diabetes-related and quality of life (SF-36) questionnaires. In addition to falls and fracture questionnaires, participants in all groups underwent blood pressure measurement, hand grip strength using Jamar hand dynamometer and timed and up go test for fall risk assessment. These tests were performed by a single research coordinator, and all measurements were taken in duplicate and averaged for analysis.

#### Imaging Procedures

All participants underwent DXA (Discovery W model for participants who were scanned prior to 2021 and Discovery Horizon after 2021) for distal 1/3rd radius, hip, and lumbar spine, as well quantitative CT (QCT) scan of hip and lumbar spine (L1 and L2). A single technician performed all DXA measurements. All QCT scans were calibrated using the Mindways Model 3 phantom system (Mindways Software, Austin, TX), which contains solid reference materials of known mineral density (effective K₂HPO₄ densities ranging from − 50 mg/cc to 375 mg/cc; 0.4% precision per manufacturer specifications). Similarly, DXA were calibrated daily per manufacture recommendation. The least significant change (LSC) for DXA bone density at the lumbar spine and at the hip was 0.069 and 0.088 gm/cm^2^, respectively. All QCT scans were read, and FE analysis was carried out by a single person (JPT). The Philips Gemini TF TOF 64 system (CT) was used for finite element (FE) analysis and bone quality measurements at the proximal hip and lumbar spine. CT scans were acquired using the following parameters: 120 kVp, helical scan mode, 380 mm × 380 mm field of view (512 × 512 matrix), and 1 mm slice thickness. The resulting voxel dimensions were 0.7422 mm × 0.7422 mm × 1 mm. CT scans were calibrated using five Mindways material phantoms for conversion to QCT scans according to the manufacturer’s standards (Mindways Software inc., Texas). QCT Pro (Mindways Software inc., Texas) software was used to determine the morphological and densitometric parameters across the left proximal femur. As there was biomechanical symmetry across the femurs, only the left femur was analyzed [16, 17]. The intertrochanteric, trochanteric, total hip, and femoral neck regions were analyzed (defined automatically using QCT Pro software). Variables analyzed included compartment volumetric bone mineral density (vBMD), mass, and volume for the cortical bone component, trabecular bone component, and total bone component in each region. In addition, cortical analysis of the cross-sectional femoral neck measured BMD, cross-sectional area (CSA), mass, volume, cross-sectional moment of inertia (CSMI), density-weighted cross-sectional moment of inertia (DWCSMI), section modulus, and maximum and minimum cortical thickness.

#### FE Analysis

An in-house MATLAB program was used to convert the brightness of each voxel in the previously collected QCT image of the proximal femur to the Young’s modulus. The conversion from the QCT density to Young’s modulus followed the methodology of Morgan et al. (2003) [18]. The resulting RAW file, which contained the calibrated image, was used to create 3-dimensional computational reconstructions of the left proximal femur in Simpleware (Synopsys, inc., California). These reconstructions were segmented into FE models using uniform 1.5 mm (for femoral models) or 1 mm (for vertebral models) octahedral elements, and material properties based on the quantitative density values of the qCT were calculated and applied to each element [19, 20]. The femoral models were oriented in two positions, standing and falling, corresponding to their physiological positions in vivo. In the standing configuration, the femoral canal was oriented vertically along the sagittal plane and at 70° above the transverse plane. In the falling configuration, the femoral neck was oriented at 15° to the frontal plane and 10° below the transverse plane. Vertebral models were oriented axially, and spinous geometry was removed, leaving only the vertebral bodies for analysis. Polymethyl methacrylate (PMMA) caps were added to the areas of force application in all models to allow uniform loading. Femoral models were then subjected to preliminary analysis for each position to determine which elements were in compression and tension, and their material properties were updated accordingly. To predict femoral fracture force models were compressed to either 4% of the femoral neck length or 4% of the femoral model height along the infero-superior axis for the standing and falling configurations, respectively. Vertebral bodies had a downward displacement equal to 2% of the overall vertebral body height applied to the superior border to the vertebral body model. The femoral fracture forces were estimated as the total force required to compress the model, based on the distances specified above. Predicted vertebral fracture force was estimated as the total force required to compress the model was multiplied by 0.34 [21].

#### Statistical Analysis

There was no formal sample size or power analysis as this was a pilot study. Data are presented as mean and standard deviation for normally distributed continuous data or as a number and percentage for categorial data. Demographic and radiological features were compared between the three groups using Chi-Square test for categorical variables and ANOVA for continuous variables. The Kruskal Wallis test was used to compare median DXA T-scores and Z-scores between the three groups. *P* < 0.05 was considered statistically significant.

## Methods for Literature Search

We searched PubMed with two search terms: “Hypophosphatasia [tiab] AND Diabetes [tiab]” and Hypophosphatasia [title] AND prevalence [title]. Only human studies reported in English were reviewed. We did not restrict the literature search to the last five years given HPP is a rare disease and its prevalence in asymptomatic general population is less studied. Using the first search term, we found 7 articles, but none were relevant to our objective, which was evaluating prevalence of HPP in patients with diabetes (either type 1 or type 2 diabetes). Using the second search term, we found 12 research articles. We reviewed their full text and relevant references of those articles were reviewed as well. A total of 14 articles that provided information on prevalence of potential HPP are detailed in Table 5 and reviewed here.

## Results

### Results of the Pilot Study

Of 1723 non-pregnant adults with T1D attending the Barbara Davis Center for Diabetes in 2020, EMR search followed by manual review of medical records identified 18 adults with potential HPP defined as at least two ALP levels < 35 U/L and no identifiable secondary cause for low ALP levels (1.04%). Of these, 12 participants consented and 10 completed the study. Three of these participants had pathologic genetic mutation in the ALP gene (0.17%) and 6 participants had elevated B6 levels (0.35%) (Supplementary Table 1). 70% of potential HPP participants reported at least one fracture in their lifetime with 60% reporting incomplete or pseudo-fractures and 30% had delayed fracture healing (Supplementary Table 2). Only 30% of participants were told by their providers that they had low ALP levels.

The baseline demographic and clinical characteristics of adults with T1D and potential HPP, adults with T1D without HPP, and adults without diabetes are provided in Table 1. Participants with potential HPP were younger than both adults with T1D and adults without diabetes (44 ± 7 vs. 49 ± 2 vs. 53 ± 5 years, *p* = 0.003) and most participants were female (HPP + T1D: 70%, T1D: 100%, control: 70%). Potential HPP participants had a shorter duration of diabetes, better diabetes control (lower HbA1c), and lower BMI compared to participants with T1D Table 1.

**Table 1:** Baseline characteristics of the study participants

	HPP + T1D (*N* = 10)	T1D (*n* = 10)	Controls (*n* = 10)	*P*-value
Age at enrollment (years)	44 ± 7	49 ± 2	53 ± 5	0.003
Sex, F %	70%	100%	70%	0.06
Postmenopausal female, % postmenopausal duration(years)	0% N/A	44% 11 ± 6	33% 9 ± 3	0.06 0.62
Smoking Current (%) Ever (%)	0 20	0 22	12.5 12.5	0.61
Alcohol > 3 drinks (%)	0	10	0	0.32
Diabetes duration (years) T1D before age 20	26 ± 6 10%	38 ± 8 10%	N/A	0.002 1.0
BMI (kg/m2)	25.9 ± 4.3	30.7 ± 6.9	28.4 ± 3.5	0.13
HbA1c at enrollment (%)	6.3 ± 0.6	7.0 ± 0.7	5.4 ± 0.3	< 0.0001
Timed up and go (seconds)	7.9 ± 0.8	7.9 ± 1.0	8.1 ± 1.1	0.84
Hand Grip Strength Left (lbs) Right (lbs)	67 ± 15 76.8 ± 19.3	57 ± 2 63.2 ± 9.9	70 ± 27 78.7 ± 29.1	0.30 0.25
SBP (mmHg)	112 ± 7	121 ± 10	124 ± 16	0.06
DBP (mmHg)	76 ± 5	73 ± 4	80 ± 8	0.047
Any fractures, %	70%	60%	50%	0.66
% with major osteoporotic fracture (fracture of hip, spine, wrist, or shoulder)	0	0	0	N/A
SF-36	69 ± 17	74 ± 15	71 ± 19	0.87

*HPP* hypophosphatasia, *T1D* type 1 diabetes, *BMI* body mass index, *SBP* systolic blood pressure, *DBP* diastolic blood pressure, *SF* short form survey, score ranges from 0 to 100 with higher score indicates better health, lbs; strength in pounds,

There were no differences in the DXA bone density at distal 1/3rd radius, left total hip, femoral neck or lumbar spine between potential HPP, T1D and controls. However, numerically, DXA BMD was modestly higher (without statistical significance) among potential HPP participants than T1D participants [Table 2]. Similarly, there was no statistically significant difference in QCT-based bone density or structure between three groups Table 3. Additionally, there were no differences in FE derived fracture force at the hip or lumbar spine between groups Table 4.

### Results of the Literature Search

We did not find any study that evaluated prevalence of HPP or low ALP levels among people with diabetes. We did not find any literature on the effects of two diseases (HPP and T1D) on skeletal phenotyping and bone quality. Hence, we believe that our pilot study was the first to evaluate the burden of two diseases and their clinical and radiological characteristics.

The literature search on use of EMR to identify potential HPP is summarized in Table 5. In general, the prevalence of low ALP levels when searching EMR is different population is varied from 0.1 to 5.5% [3, 4, 22–33]. The differences in prevalent low ALP levels were due to inherent differences in the population studies, sample size and EMR methods used. Despite higher levels of low ALP levels in various studies, the prevalence of potential HPP based on persistent low ALP with exclusion of secondary causes of low ALP levels in presence of some clinical or radiological sign or symptoms is low and ranged between 0.06 and 1% [3, 4, 22–33]. Interestingly, most studies had low acceptance of participation (or low recruitment) of people with low ALP levels (generally < 20–30% of potential HPP). Similarly in our study 10 people out of 18 adults with T1D and potential HPP agreed to take part in the study (~ 50%). This may be due to low awareness about the HPP disease and asymptomatic nature of the disease in a patient who is otherwise healthy may not feel incentivized to participate in the HPP research.

## Summary

This is the first study to estimate the potential prevalence of HPP in adults with T1D. We found potential prevalence of HPP was ~ 1% based on persistent low ALP level, 0.35% based on both low ALP and high serum vitamin B6 level, and 0.17% based on genetic testing. These rates are almost similar to that is being reported in the literature [3, 4, 22–33]. The ALP gene is located on chromosome 1 (1p36.12). The most common genes implicated in T1D are human leukocyte antigen (HLA) DR (DR 3 and 4) and DQ (DQ 2 and 8) which are located on chromosome 6p21. We believe that HPP and T1D are probably unrelated and hence, the prevalence of HPP in people with T1D would be similar to that of the general population.

The fracture burden was higher among potential HPP participants (70%), and the majority reported incomplete fracture or delayed fracture healing. We did not administer HPP disease burden questionnaires to participants with T1D and controls without diabetes and hence, it is difficult for us to compare fracture burden between potential HPP and T1D participants.

We did not find any significant differences in bone density measured by DXA or QCT-based bone density between the three groups. Our pilot study suggests that bone density or currently used methodologies in research tools such as QCT or FE measured bone strength do not aid in fracture risk estimation in HPP and T1D. Number of studies have reported higher DXA BMD among adults with HPP suggesting compromised mineralization leading to higher BMD. [5, 34–36] Two studies have evaluated bone microarchitecture using high resolution peripheral QCT (HR-pQCT) of distal radius and tibia in patients with HPP [37, 38]. Both studies have showed tendency towards reduced trabecular volumetric density, increased trabecular separation at the distal radius, however, these changes were not statistically significant in the study by Hepp et al. [38]. To our knowledge, there is no published study evaluating bone architecture at the hip and lumbar spine. It is possible that HPP may have some differential effects on axial vs. peripheral skeleton and may be affecting more of trabecular bone. Given high resolution of HR-pQCT, the changes in trabecular bone could be more pronounced than QCT. Our study and other studies are consistently suggesting that HPP is a disease of low bone quality due to impaired ALP activity and thus, imaging techniques that provide only information on density may not provide sufficient information on fracture risk. Similarly, our previous studies in T1D suggested that DXA bone density in people with T1D is similar to people without diabetes (except modestly lower femoral neck BMD especially in postmenopausal females) suggesting that bone density does not estimate true fracture risk in T1D [39, 40]. Thus, HPP and T1D have similar issue of impaired bone quality without affecting bone density.

Our study has many major clinical and research implications: First, our study confirms reports of previous studies suggesting that systematic EMR search is feasible and may yield higher prevalence of HPP. This could be an important strategy to diagnose patients with HPP who otherwise would remain undiagnosed leading to increased fracture burden among individuals as well as associated increased in health care cost. Second, since fracture risk is higher in people with T1D and it’s characterized by low bone turnover, HPP may confound fracture risk and should be ruled out in those with low serum ALP levels. Third, radiological measures may not yield much value in identifying HPP in presence of T1D. Our study also highlights potential limitations of FE-based bone strength measurements. Current FE standards were designed using normal healthy population and hence, methods calculating mechanical properties based on clinical values (such tissue density to material stiffness) may be inappropriate. This is especially true if biomechanical factors below the resolution of the imaging modality used in FE generation are not accounted for. Thus, methods for imputing impaired bone quality of HPP need to be refined, otherwise FE may not provide reliable estimates of bone strength in people with HPP.

The results of our study should be interpreted with caution as this was a pilot study without formal sample size estimates. There were only three participants with pathological mutations limiting our ability to compare clinical and radiological characteristics between those with pathological mutations and biochemical disease (Low ALP with elevated B6) without mutations. It is likely that potential HPP patients in our study may not have clinical HPP as single pathological mutations or simply low ALP with high Vitamin B6 levels alone does not constitute clinical HPP. The International Working Group was convened in 2021 and published proposed criteria for diagnosis of HPP in 2023 [41]. Unfortunately, our study was conducted before the proposed diagnostic criteria for HPP was published and hence, we did not define HPP based on these currently accepted diagnostic criteria. However, 70% of potential HPP patients reported at least one fracture with 60% reporting pseudofracture or incomplete fracture and 30–40% reporting various musculoskeletal chronic clinical problems suggest that many of these patients had clinical HPP.

In conclusion, HPP is common in adults with T1D, and EMR-based HPP case finding is feasible and may uncover a higher number of potentially undiagnosed HPP. DXA and QCT-based distal 1/3rd radius, hip and lumbar spine bone density and structure was similar between adults with T1D and potential HPP compared to adults with T1D without HPP, but this may be due to limitations in currently available methodologies.

**Table 2:** DXA-based bone density between three groups

DXA site*	HPP + T1D (*n* = 10)	T1D (*n* = 10)	Controls (*n* = 10)	*P*-value
Total hip, Left, gm/cm^2^	1.02 ± 0.15	0.96 ± 0.11	0.96 ± 0.09	0.4
Femoral neck, left, gm/cm^2^	0.89 ± 0.13	0.81 ± 0.12	0.84 ± 0.12	0.5
Total hip, T-score	0.9 (−0.3, 1.0)	0.05 (−0.6, 0.5)	−0.05 (−0.4, 0.3)	0.7
Total hip, Z-score	1.1 (−0.2, 1.3)	0.45 (−0.1, 0.9)	0.3 (0.0, 0.7)	0.7
L-L4, gm/cm^2^	1.07 ± 0.12	1.08 ± 0.15	1.12 ± 0.20	0.8
L1-L4, T-score	0.1 (−0.4, 0.2)	0 (−0.3, 0.6)	0.25 (−0.4, 1.2)	0.8
L1-L4, Z-score	0.4 (−0.1, 0.6)	0.7 (0.4, 1.2)	0.85 (0.1, 1.8)	0.5
Distal 1/3rd radius, gm/cm^2^	0.79 ± 0.07	0.70 ± 0.11	0.78 ± 0.09	0.08
Distal 1/3rd radius, T-score	1.1 (0.6, 1.4)	0.5 (0.2, 1.2)	0.9 (−0.03, 1.5)	0.5
Distal radius 1/3rd, Z-score	1.5 (0.9, 2.0)	1.1 (0.8–1.9)	1.5 (0.5, 2.1)	0.7

*We showed only left total hip and left femur as the correlation between right and left is very high. Non-dominant forearm was considered for DXA of distal 1/3rd radius.

Data shown as mean ± SD or median (IQR).

**Table 3:** QCT-based volumetric density at hip in three groups

Hip region	HPP + T1D (*n* = 10)	T1D (*n* = 10)	Controls (*n* = 10)	*P*-value
Total hip, gm/cm^3^				
Total vBMD	0.338 ± 0.07	0.339 ± 0.06	0.299± 0.05	0.2
Cortical vBMD	0.875 ± 0.09	0.880 ± 0.05	0.878 ± 0.05	0.9
Trabecular vBMD	0.158 ± 0.03	0.149 ± 0.03	0.139 ± 0.01	0.2
Femoral neck, gm/cm^3^				
Total vBMD	0.355 ± 0.07	0.345 ± 0.07	0.308 ± 0.05	0.2
Cortical vBMD	0.849 ± 0.08 ^b^	0.884 ± 0.06	0.925 ± 0.09	0.1
Trabecular vBMD	0.168 ± 0.03	0.153 ± 0.04	0.150 ± 0.02	0.3
Trochanter, gm/cm^3^				
Total vBMD	0.264 ± 0.05	0.236 ± 0.05	0.222 ± 0.04	0.2
Cortical vBMD	0.821 ± 0.02	0.818 ± 0.07	0.911 ± 0.03	0.4
Trabecular vBMD	0.151 ± 0.03	0.141 ± 0.02	0.137 ± 0.02	0.3
Intertrochanter, gm/cm^3^				
Total vBMD	0.367 ± 0.08	0.396 ± 0.06	0.339 ± 0.05	0.2
Cortical vBMD	0.910 ± 0.01	0.895 ± 0.05	0.874 ± 0.04	0.6
Trabecular vBMD	0.158 ± 0.03	0.154 ± 0.03	0.138 ± 0.02	0.2

^a^*p*<0.05 for HPP+T1D vs. T1D; ^b^*p*<0.05 for HPP+T1D vs. Controls; ^c^*p*<0.05 for T1D vs. Controls

**Table 4:** FE- based bone strength between three groups

	HPP + T1D (*n* = 10)	T1D (*n* = 10)	Controls (*n* = 10)	*P*-value
FE-derived fracture force at femoral neck, standing, N	13,048 ± 1376	12,279 ± 2858	12,721 ± 3574	0.8
FE-derived fracture force at femoral neck, falling, N	5553 ± 1123	4993 ± 1240	4641 ± 1283	0.3
FE-derived fracture force at L1, N	7040 ± 1071 ^**b**^	6492 ± 1567	5701 ± 1287	0.09
FE-derived fracture force at L2, N	7613 ± 1051	6944 ± 1629	6451 ± 1327	0.2

N; newton, FE; finite element

^b^*p* < 0.05 for HPP + T1D vs. Controls.

**Table 5:** Summary of published studies reporting prevalence of potential HPP

First author (ref)	Country	Study design	Population	Defining low ALP values^1^	Prevalence of low ALP level^2^	Prevalence of potential HPP^3^	Prevalence of genetically proven HPP^4,5,6^
McKiernan FE (22)	USA	Retrospective EMR review	Outpatient clinic	ALP ≤ 30 in absence of known causes	NA	NA	42/50 (84%)
McKiernan FE (3)	USA	Retrospective EMR review	Outpatient clinic	Screening: ALP < 40 Confirmatory: at least two ALP ≤ 30	1.2%	1/1544 (0.06%)	NA
Riancho-Zarrabeitia (23)	Spain	Retrospective review of Laboratory database	laboratory data base	Screening: ALP < 40 Confirmatory: <26 and no value > 40.	12,546/ 500,000 (2.5%)	50/8758 (0.6%)	21/42 (50%)
Larid G (24)	France	Retrospective analysis of laboratory database	Hospitalized adults in rheumatology and internal medicine departments, multicenter.	At least two ALP ≤ 35	664/56,321 (1.2%)	182/56,321 (0.3%)	11/18 (61%)
Maman E (25)	France	Retrospective, review of Laboratory database	Laboratory database	Screening: ALP < 30 Confirmatory: ALP < 30 and no ALP > 40	241/48,755 (0.16%)	62/48,755 (0.13%)	NA
Hepp N (26)	Denmark	Retrospective EMR review	Endocrine clinic	At least two ALP ≤ 35 and no ALP ≥ 35	NA	51/26,121 (0.2%)	13/24 (54%)
García-Fontana C (27)	Spain	Retrospective review of Laboratory database	Laboratory database	Screening: ALP < 40 Confirmatory: ALP ≤ 30	1907/76,083 (2.5%)	56/76,083 (0.07%)	7/16 (43%)
Schmidt T (28)	Germany	Retrospective review of Laboratory database	Laboratory database	ALP < 30	585,287/6,918,126 (8.5%)	1/195 (0.5%)*	NA
Vieira LHR (29)	Brazil	Retrospective review of Laboratory database	Laboratory database of hospital	At least two ALP < 40	NA	12/1049 (1.1%)	NA
Feurstein J (30)	Austria	Retrospective EMR review	Outpatient rheumatology clinic	ALP ≤ 40	524/ 9522 (5.5%)	73/9522 (0.8%)	13/23 (56%)
Koehler K (4)	USA	Retrospective	Podiatry clinic, single center presented with metatarsal stress fracture	ALP < 32–38 depending on reference value of the lab	13/937 (1.4%)	NA	2/8 (25%)
Alonso N (31)	UK	Retrospective EMR review	Osteoporosis clinic	At least 2 ALP ≤ 40	16/3285 (0.5%)	NA	14/16 (87.5%)
Calmarza P (32)	Spain	Retrospective review of Laboratory database	Laboratory database	Screening; ALP < 30 Confirmatory: ALP < 30 and never > 35	NA	NA**	NA
Quinn HB (33)	USA	Retrospective EMR review	Endocrine Clinic	ALP ≤ 40	315/20,000 (1.6%)	10/20,000 (0.05%)	NA

NA; data not available or prevalence could not be calculated due to missing information. ALP; alkaline phosphatase (reported as IU/L)

^a^Most studies have reported EMR search using two different methods; initial screening using a relaxed cut-off values and second, more stringent cut-off and/or manual chart review.

^b^ prevalence of low ALP values is calculated as number of people with low ALP levels based on initial screening methods divided by total sample size or population.

^c^ potential HPP is defined as persistent low ALP levels (as defined by the studies) in absence of other causes and/or with clinical signs or symptoms and/or radiological findings and/or genetic testing. There were high variations on how persistent low ALP levels and potential HPP defined across the studies.

^d^ There were low enrollment reported in most studies (i.e. number of people participated in the study were ~20-30% of total eligible participants with suspected HPP).

^e^The values reported as number of people with positive genetic testing/ number of people tested for genetic testing (%).

^f^Not all reported genetic mutations in various studies were pathological.

*defined as AL<30 and elevated PLP level.** 21 out of 589 individuals with low ALP had potential HPP. Total population sample size was reported.

## Supplementary Information

Below is the link to the electronic supplementary material.

ESM 1 (DOCX 18.3 KB) 

## Data Availability

De-identified aggregated data will be available upon written request to corresponding author (VNS).
