# Supplementary material for: Hypophosphatasia and Type 1 Diabetes: A Pilot Study and Review of Literature
Source: Curr Osteoporos Rep. 2025 Oct 9;23(1):42. doi: 10.1007/s11914-025-00938-x (PMC12511211; doi:10.1007/s11914-025-00938-x)
Supplement: Supplementary file 1 — (DOCX 18.3 KB) [file 11914_2025_938_MOESM1_ESM.docx]

**Supplementary Table 1**

| Subject ID | DNA variants | Interpretation | ALP level (U/L)^#^ | Vitamin B6 level (nmol/L) |
| --- | --- | --- | --- | --- |
| 1 | c407 G>A, p.Arg136His | Pathogenic | 36 | 142.5 |
| 2 |  | Negative | 31 | 133.6 |
| 3 |  | Negative | NA | NA |
| 4 | c4346 G>A, p.Glu148Lys | Pathogenic | 21 | 249.9 |
| 6 |  | negative | 31 | NA |
| 7 |  | Negative | 29 | 824.6 |
| 8 |  | NA | NA | 125 |
| 10 |  | negative | NA | 33.5 |
| 11 |  | negative | 34 | 193.8 |
| 12 | c.407G>A, p.Arg136His | Pathogenic | 24 | 107.5 |

NA; report not available.

Normal reference range for total ALP (for age >18 years for both sexes) was 39-117 U/L and 20-125 nmol/L for vitamin B6.

# All patients had at least two serum ALP levels <35 U/L recorded from the EMR. The reported ALP in this table is measured at the time of screening visit.

**Supplementary Table 2: Responses to HPP questionnaire among potential HPP and T1D participants (n=10)**

| Questions | Yes (%) |
| --- | --- |
| Do you know what hypophosphatasia is? | 3 (30%) |
| Have you ever been told that you have low ALP, a laboratory test routinely done for annual blood work? | 3 (30%) |
| Have you had premature tooth loss as a child? | 0% |
| Have you ever had spontaneous tooth loss without any trauma? | 0% |
| Have you ever been diagnosed with tooth abscess? | 0% |
| Have you ever been diagnosed with osteopenia or low bone density? | 0% |
| Have you ever been diagnosed with osteoporosis? | 0% |
| Have you ever had bone deformity as a child or as an adult? | 1 (10%) |
| Have you ever fractured a bone? | 7 (70%) |
| When did your first fracture occur? | Childhood (10%)  Adolescent (40%)  Adult (20%) |
| Have you had incomplete or pseudofracture? Incomplete is defined as the bone does not break completely. | 60% |
| Have you had any fracture that took longer time (>8 weeks) to heal? | 30% |
| How long did it take for fracture to heal? | 2-3 month (20%)  3-6 month (10%) |
| Do you experience bone pains? | 10% |
| Is your bone pain severe enough to limit your activities? | 10% |
| Do you have muscle pain? | 30% |
| Do you have joint pains? | 40% |
| Have been diagnosed with calcium deposits in your joints or muscle? | 10% |
| Have you ever been diagnosed with kidney stones? | 0% |
|  |  |
